# Surface Current in “Hotspot” Serves as a New and Effective Precursor for El Niño Prediction

**DOI:** 10.1038/s41598-017-00244-2

**Published:** 2017-03-13

**Authors:** Jianing Wang, Youyu Lu, Fan Wang, Rong-Hua Zhang

**Affiliations:** 1 Key Laboratory of Ocean Circulation and Waves, Institute of Oceanology, Chinese Academy of Sciences, Qingdao, China; 2 Laboratory for Ocean and Climate Dynamics, Qingdao National Laboratory for Marine Science and Technology, Qingdao, China; 3 Bedford Institute of Oceanography, Fisheries and Oceans of Canada, Dartmouth, Nova Scotia Canada; 4 University of Chinese Academy of Sciences, Beijing, China

## Abstract

The El Niño and Southern Oscillation (ENSO) is the most prominent sources of inter-annual climate variability. Related to the seasonal phase-locking, ENSO’s prediction across the low-persistence barrier in the boreal spring remains a challenge. Here we identify regions where surface current variability influences the short-lead time predictions of the July Niño 3.4 index by applying a regression analysis. A highly influential region, related to the distribution of wind-stress curl and sea surface temperature, is located near the dateline and the southern edge of the South Equatorial Current. During El Niño years, a westward current anomaly in the identified high-influence region favours the accumulation of warm water in the western Pacific. The opposite occurs during La Niña years. This process is seen to serve as the “goal shot” for ENSO development, which provides an effective precursor for the prediction of the July Niño 3.4 index with a lead time of 2–4 months. The prediction skill based on surface current precursor beats that based on the warm water volume and persistence in the subsequent months after July. In particular, prediction based on surface current precursor shows skill in all years, while predictions based on other precursors show reduced skill after 2002.

## Introduction

**Figure 1:**
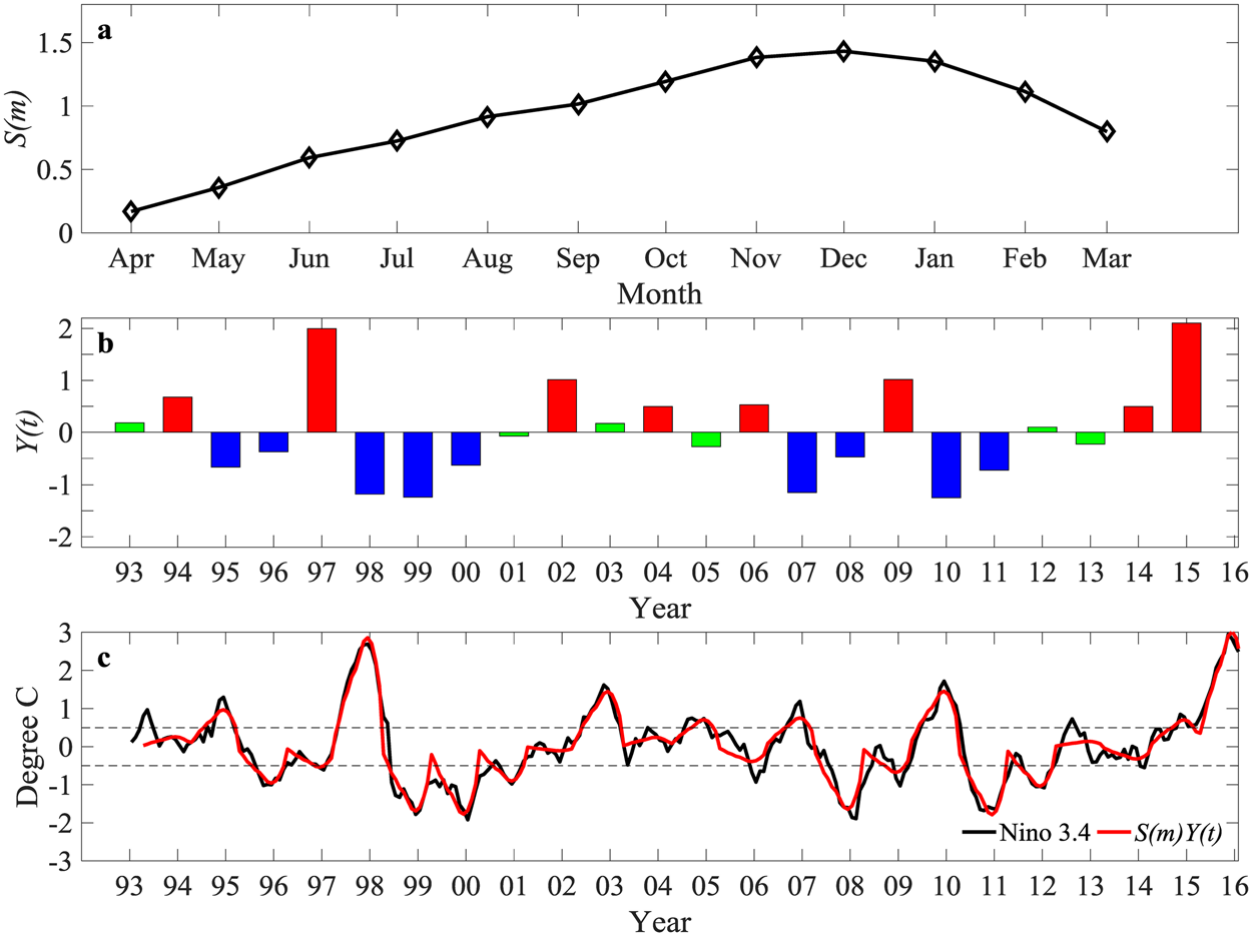
Phase locking of the Niño 3.4 index to the seasonal cycle. (**a**) Calendar-year structure function *S*(*m*) (*m* = 1, 2, … 12 correspond to April, May, … next March) and (**b)** annual amplitude (*Y*(*t*)) of the first EOF mode of the Niño 3.4 index as a function of years from 1993 to 2015. The first EOF mode explains 92% of the total variance. In (**b)**, red and blue bars denote El Niño or La Niña years, defined according to *S*(*m*)*Y*(*t*) > 0.5 °C or *S*(*m*)*Y*(*t*) < −0.5 °C persisting for at least three months, respectively; green bars denote the remaining neutral years. (**c**) Monthly time series of the Niño 3.4 index (black) and *S*(*m*)*Y*(*t*) (red), the two having a correlation of 0.96. The figure is plotted using MATLAB R2015b (http://www.mathworks.com/).

The past decades have seen significant progress in understanding the physics and predictability of the El Niño and Southern Oscillation (ENSO)^1–5^ (e.g., a recent review by Clarke^6^). In particular, a fairly robust phase-lock of interannual variability to the seasonal cycle has been revealed^7^. This is demonstrated by applying an Empirical Orthogonal Function (EOF) analysis to the Niño 3.4 index (Fig. 1). The annual amplitude function defines an El Niño, La Niña or neutral year; and the evolution of an ENSO event follows the calendar-year structure function. The ENSO’s persistence from July to February in the following year has a high level of predictability. One main and remaining challenge is the so-called spring predictability barrier (SPB)^6^. That is, the skill of prediction made from April/May to July (i.e., the transition through sparing) is lower than in other months for all dynamical and statistical forecasting models^2^.

Statistical models based on previously identified precursors, including the equatorial Pacific Warm Water Volume (WWV)^8–10^ and Indo-Pacific equatorial wind^11, 12^, show reduced prediction skill for ENSO after 2002^6^. This has been attributed to the weaker ENSO amplitude, more frequent shifts between El Niño and La Niña, and a tendency toward more central Pacific than eastern Pacific El Niños^2, 7, 13–15^. Because of its role in the zonal displacement of the Warm Pool, zonal surface current anomaly (*U*) in the equatorial Pacific has been identified as a potential additional precursor. An index based on *U* averaged over the Niño 3.4 region (5°N–5°S, 170–120°W) shows a precursor property similar to the WWV, but with a slightly lower precursor correlation^6^.

**Figure 2:**
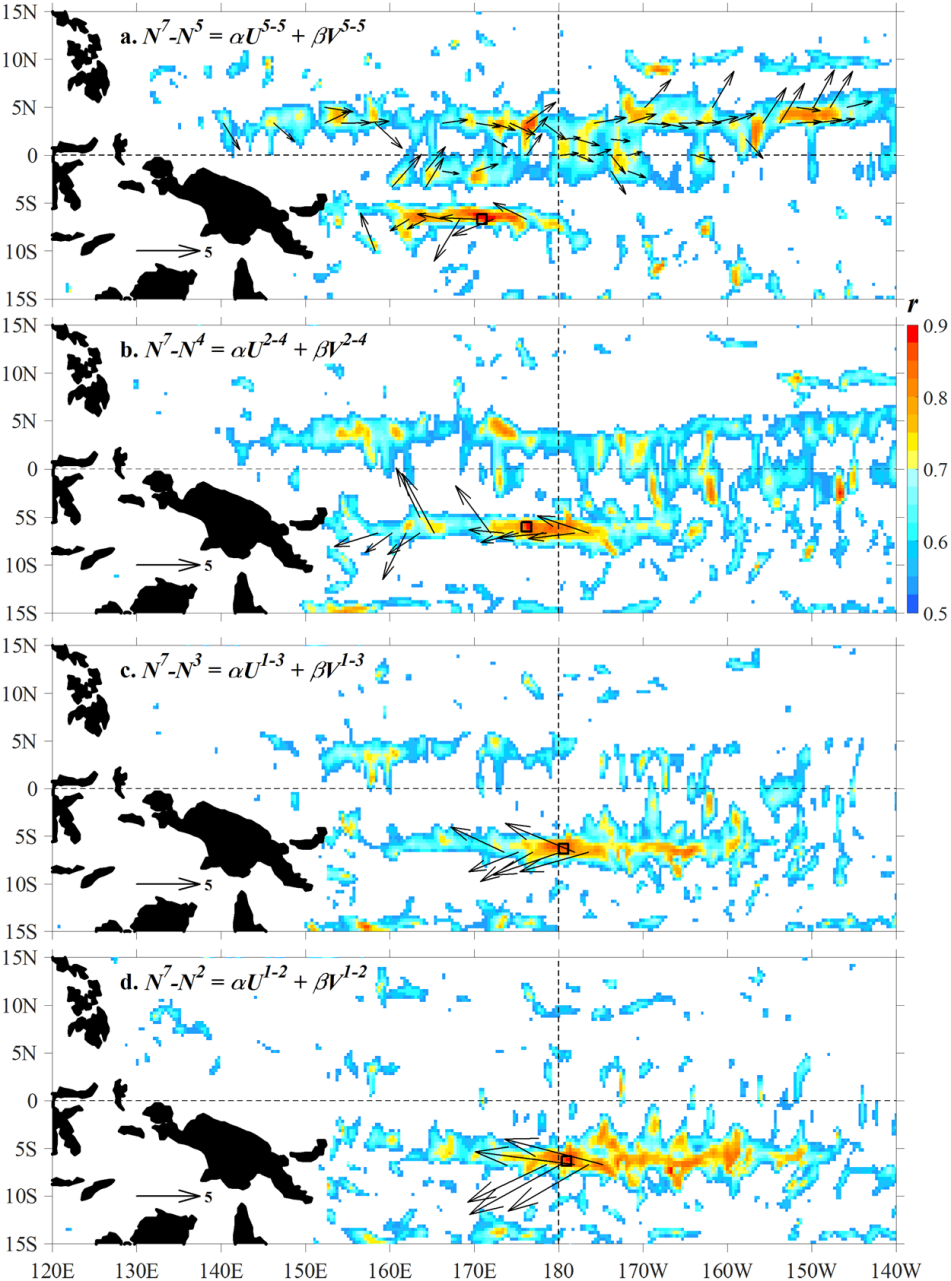
Regression relationships between the Niño 3.4 index and surface currents. Colour shading: correlations (*r*) between annual time series of *N* ^*7*^-*N* ^*t*^ and regressed *αU* ^*i*−*t*^ + *βV* ^*i*−*t*^ that are significant at the 0.01 level. Vectors: regression coefficients (*α*, *β*). *N* ^*7*^ and *N* ^*t*^ denote the Niño 3.4 index in July and in month *t*, respectively; (*U* ^*i*−*t*^, *V* ^*i*−*t*^) denote surface currents averaged from month *i* to *t*. (**a**) *t* = *i* = 5 (May). (**b**) *t* = 4 (April) and *i* = 2 (February). (**c**) *t* = 3 (March) and *i* = 1 (January). (**d**) *t* = 2 (February) and *i* = 1 (January). The figure is plotted using MATLAB R2015b (http://www.mathworks.com/). The maps in this figure are generated by MATLAB R2015b with Basemap (a mapping package, http://stockage.univ-brest.fr/~scott/MatLab/basemap.m).

In this study, we show that the ENSO prediction skill across the SPB using surface currents can be significantly improved by identifying “hotspots” of correlation between the Niño 3.4 index and surface currents (from satellite remote sensing) over the whole tropical Pacific region. This is achieved by undertaking a multivariate regression analysis^16^ (Methods). The analysis reveals regions where surface current variability influences ENSO, and the directions of these currents. Figure 2 presents the results of four regressions “trials”. (1) A 2-month-lead “prediction” of the July Niño 3.4 index (denoted as *N* ^*7*^) is based on surface current anomalies in May. It shows two hotspots: one straddling the equator between 170°E and 140°W, and the other centered at 6°S between 160°E and the dateline. An increase of the Niño 3.4 index is associated with strengthening of the eastward velocity in the northern and the westward velocity in the southern hotspots, respectively. (2) Two similar hotspots are found for a 3-month-lead “prediction” from April, based on surface currents averaged from February to April. In this case, the area of the northern hotspot shrinks, while the southern one moves eastward. (3) A 4-month-lead “prediction” from March is based on surface currents averaged from January to March. In this case, the northern hotspot nearly vanishes, while the southern one moves further eastward. (4) A 5-month-lead “prediction” from February is based on surface currents averaged from January to February. In this case, the northern hotspot completely vanishes, while the southern hotspot expands in the meridional direction. The eastern boundary of the southern hotspot is located at the dateline, 170°W, 160°W and 150°W for prediction lead times of 2, 3, 4 and 5 months, respectively.

**Figure 3:**
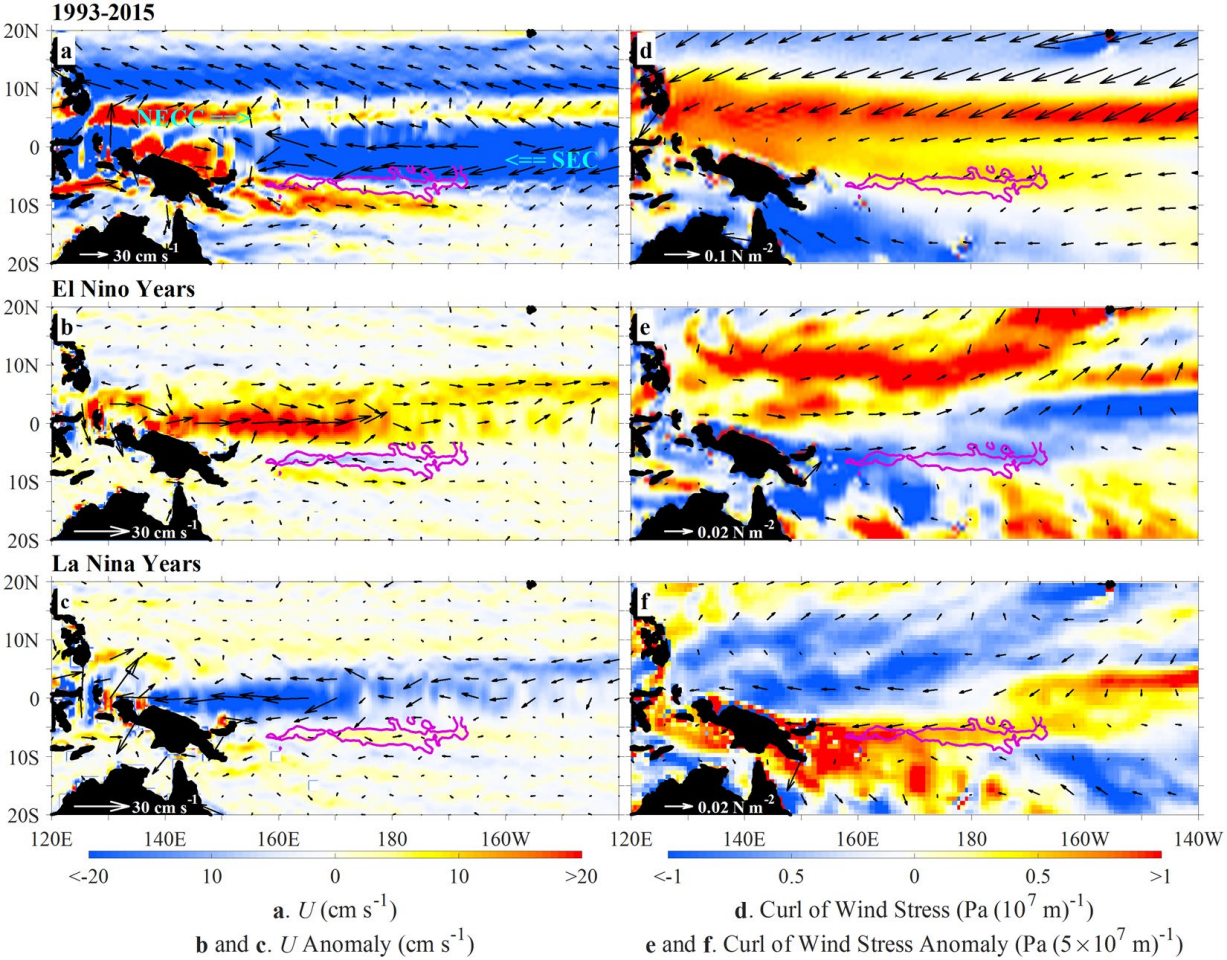
Surface current and wind stress curl averaged over February-April during 1993–2015. **Left:** surface current (vectors) and the zonal velocity (color shading). **Right:** wind stress (vectors) and its curl (color shading). **Top, Middle and Bottom**: the time-mean over all the years, and anomalies during El Niño and La Niña years, respectively. Pink contours: correlation *r* = 0.65 between *N* ^*7*^-*N* ^*4*^ and regressed surface currents over the southern hotspot shown in Fig. 2b. The figure is plotted using MATLAB R2015b (http://www.mathworks.com/). The maps in this figure are generated by MATLAB R2015b with Basemap (a mapping package, http://stockage.univ-brest.fr/~scott/MatLab/basemap.m).

**Figure 4:**
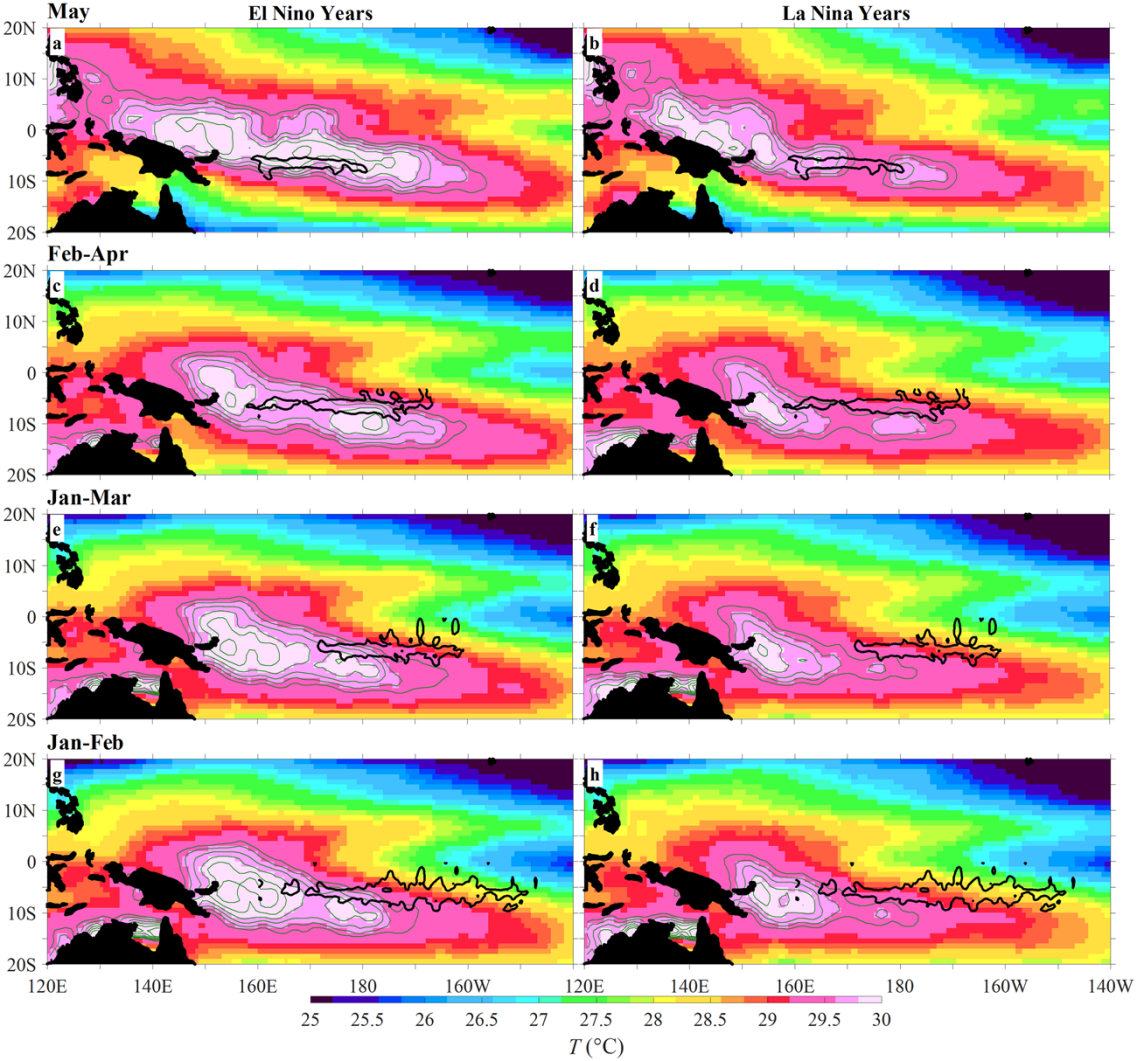
SST averaged over El Niño (left) and La Niña (right) years during 1993–2015. Results in (from **top to bottom**) May, February-April, January-March, and January–February. Green contours: isotherms from 29.5 to 29.9 °C with an interval of 0.1 °C. Black contours: correlation *r* = 0.65 between *N* ^*7*^-*N* ^*t*^ and regressed surface currents over the southern hotspot shown in Fig. 2a, b, c and d. The figure is plotted using MATLAB R2015b (http://www.mathworks.com/). The maps in this figure are generated by MATLAB R2015b with Basemap (a mapping package, http://stockage.univ-brest.fr/~scott/MatLab/basemap.m).

The locations of hotspots identified by the regression analysis hint at the role played by currents in the displacement of warm water. Figures 3 and 4 show the hotspot positions relative to two major branches of surface currents, and the distributions of sea surface temperature (SST) in the tropical Pacific. The northern hotspot lies in the interleaving area of the eastward-flowing North Equatorial Countercurrent (NECC) and westward-flowing South Equatorial Current (SEC). During El Niño years, the enhanced NECC and the weakened SEC facilitate the eastward migration of the Warm Pool, as revealed previously^17–19^. The southern hotspot is located at the southern edge of the SEC (Fig. 3), and between two cores of maximum SST (Fig. 4). During El Niño years, the westward current in the southern hotspot intensifies, resulting in displacement of warm water from east to west. The opposite situation happens in La Niña years. Previous studies have revealed that the accumulation of warm water in the western Pacific is a necessary precondition for the onset of El Niño^20, 21^. Thus, the currents in the southern hotspot may be particularly useful for ENSO prediction across the SPB.

The zonal current anomaly in the southern hotspot can be related to the anomalous curl of wind stress. We take averages from February to April (right panels of Fig. 3) as an example. The mean wind stress curl is positive over the southern hotspot and to its north, and negative to the south (Fig. 3d), corresponding to downwelling and upwelling, respectively, in the southern hemisphere. During El Niño years, the curl anomaly is generally negative near the southern hotspot, weakens the downwelling to the north and enhances upwelling to the south. This favours the shaping of the SST distribution with the maximum SST being located just to the south of the hotspot (Fig. 4c). This leads to an increase in the westward current in the southern hotspot (Fig. 3b). Note that to both the north and south of this hotspot the anomalous zonal currents are eastward, so enhancement of the westward displacement of warm water occurs only in this very localized southern hotspot during the transition stage of the ENSO cycle. The situation is reversed during La Niña years.

**Figure 5:**
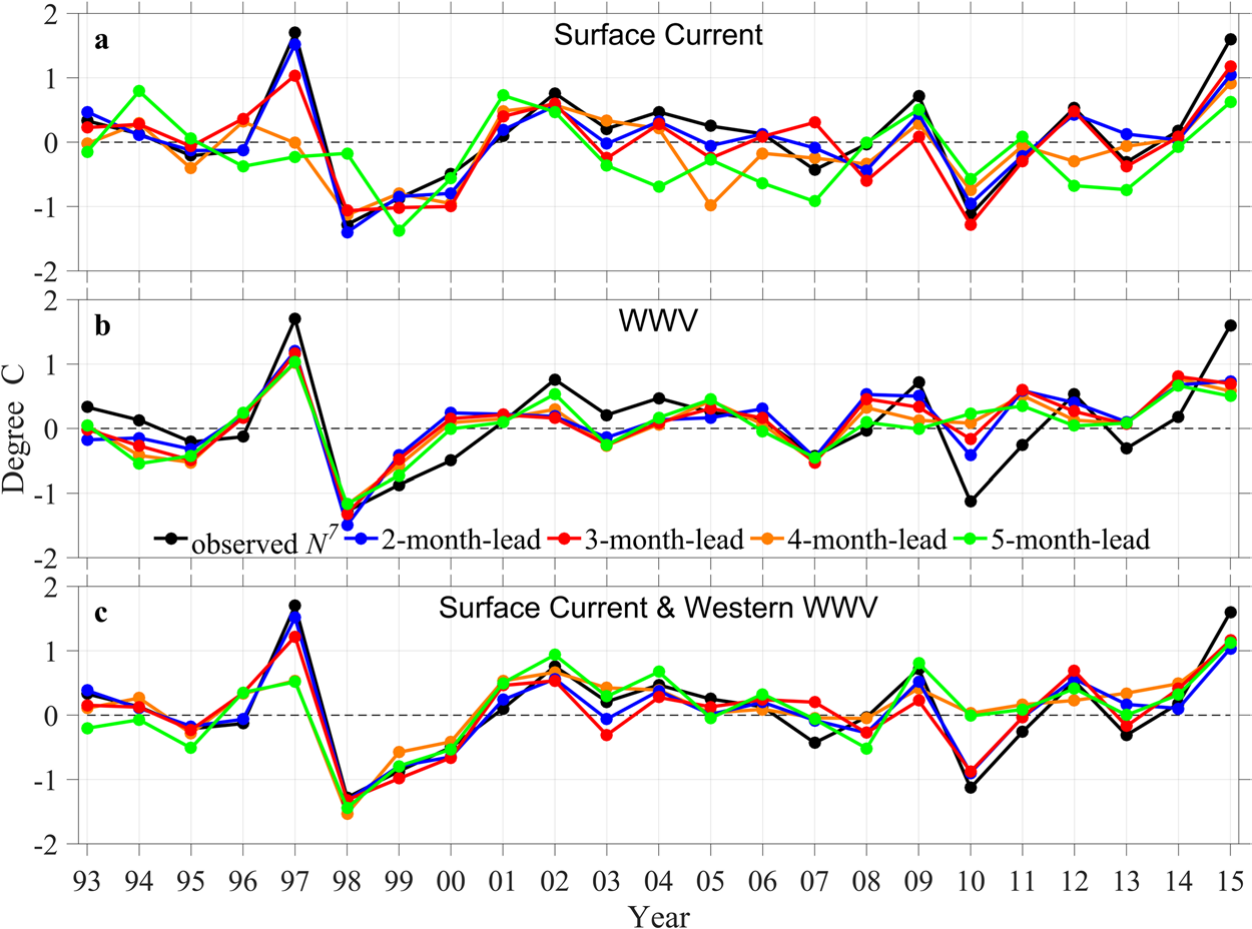
Retrospective prediction of the July Niño 3.4 index (*N* ^*7*^). Annual time series of *N* ^*7*^ from observation (black) and prediction using (**a)**, surface currents (*U* and *V*), (**b**) total WWV and (**c)**, both surface current and western WWV. Blue, red, orange and green lines correspond to predictions with lead time of 2, 3, 4 and 5 months, respectively. Surface currents are taken from locations in the southern hotspot denoted by black squares in Fig. 2 and given in Table 1. The figure is plotted using MATLAB R2015b (http://www.mathworks.com/).

The prediction of *N* ^*7*^ can be formulated through a multivariate regression to various precursors in the spring transition stage, including surface currents in the southern hotspot, and the total and western WWV (Methods). The prediction skill is illustrated in Fig. 5 and summarized in Table 1. If only surface currents are used, the correlations between the observed and predicted *N* ^*7*^ with lead times of 2, 3, 4 and 5 months are 0.95, 0.87, 0.68 and 0.64, respectively. If only the total WWV are used, the corresponding correlations are 0.76, 0.72, 0.67 and 0.66. Combining both the surface currents in the southern hotspot and the western WWV, the corresponding correlations are increased to 0.95, 0.90, 0.77 and 0.78, respectively.

**Table 1:** Correlation (*r*) between the Niño 3.4 index in July observed and predicted using surface current with lead time of 2 to 4 months. (*U*, *V*) are the surface current components at a location given in longitude/latitude in the southern hotspot.

Lead Time	Surface Current			Surface Currents and Western WWV
2 months in May	(*U* ^*3*−*5*^, *V* ^*3*−*5*^)	(*U* ^*4*−*5*^, *V* ^*4*−*5*^)	(*U* ^*5*−*5*^, *V* ^*5*−*5*^)	(*U* ^*5*−*5*^, *V* ^*5*−*5*^, ${W}_{W}^{{5}-{5}}$)
	*r*: 0.91 at	*r*: 0.95	*r*: 0.95	*r*: 0.95
	174.2°E, 6.0°S	172.2°E, 6.3°S	171.2°E, 6.7°S	171.2°E, 6.7°S
3 months in April	(*U* ^*2*−*4*^, *V* ^*2*−*4*^)	(*U* ^*3*−*4*^, *V* ^*3*−*4*^)	(*U* ^*4*−*4*^, *V* ^*4*−*4*^)	(*U* ^*2*−*4*^, *V* ^*2*−*4*^, ${W}_{W}^{{4}-{4}}$)
	*r*: 0.87 at	*r*: 0.79 at	*r*: 0.81 at	*r*: 0.90
	176.2°E, 6.0°S	178.6°E, 6.3°S	174.9°E, 6.3°S	176.2°E, 6.0°S
4 months in March	(*U* ^*1*−*3*^, *V* ^*1*−*3*^)	(*U* ^*2*−*3*^, *V* ^*2*−*3*^)	(*U* ^*3*−*3*^, *V* ^*3*−*3*^)	(*U* ^*1*−*3*^, *V* ^*1*−*3*^, ${W}_{W}^{{3}-{3}}$)
	*r*: 0.68 at	*r*: 0.65 at	*r*: 0.57 at	*r*: 0.77
	180.6°E, 6.3°S	180.6°E, 6.3°S	177.6°E, 7.7°S	180.6°E, 6.3°S
5 months in February	(*U* ^*12*−*2*^, *V* ^*12*−*2*^)	(*U* ^*1*−*2*^, *V* ^*1*−*2*^)	(*U* ^*2*−*2*^, *V* ^*2*−*2*^)	(*U* ^*1*−*2*^, *V* ^*1*−*2*^, ${W}_{W}^{{2}-{2}}$)
	*r*: 0.48 at	*r*: 0.64 at	*r*: 0.58 at	*r*: 0.78
	186.9°E, 6.3°S	180.9°E, 6.3°S	186.2°E, 4.7°S	180.9°E, 6.3°S

*W*_*w*_ denote the western WWV. Super-scripts on *U*, *V* and *W*_*W*_ denote averaging periods following the same definition as in Fig. 2.

**Figure 6:**
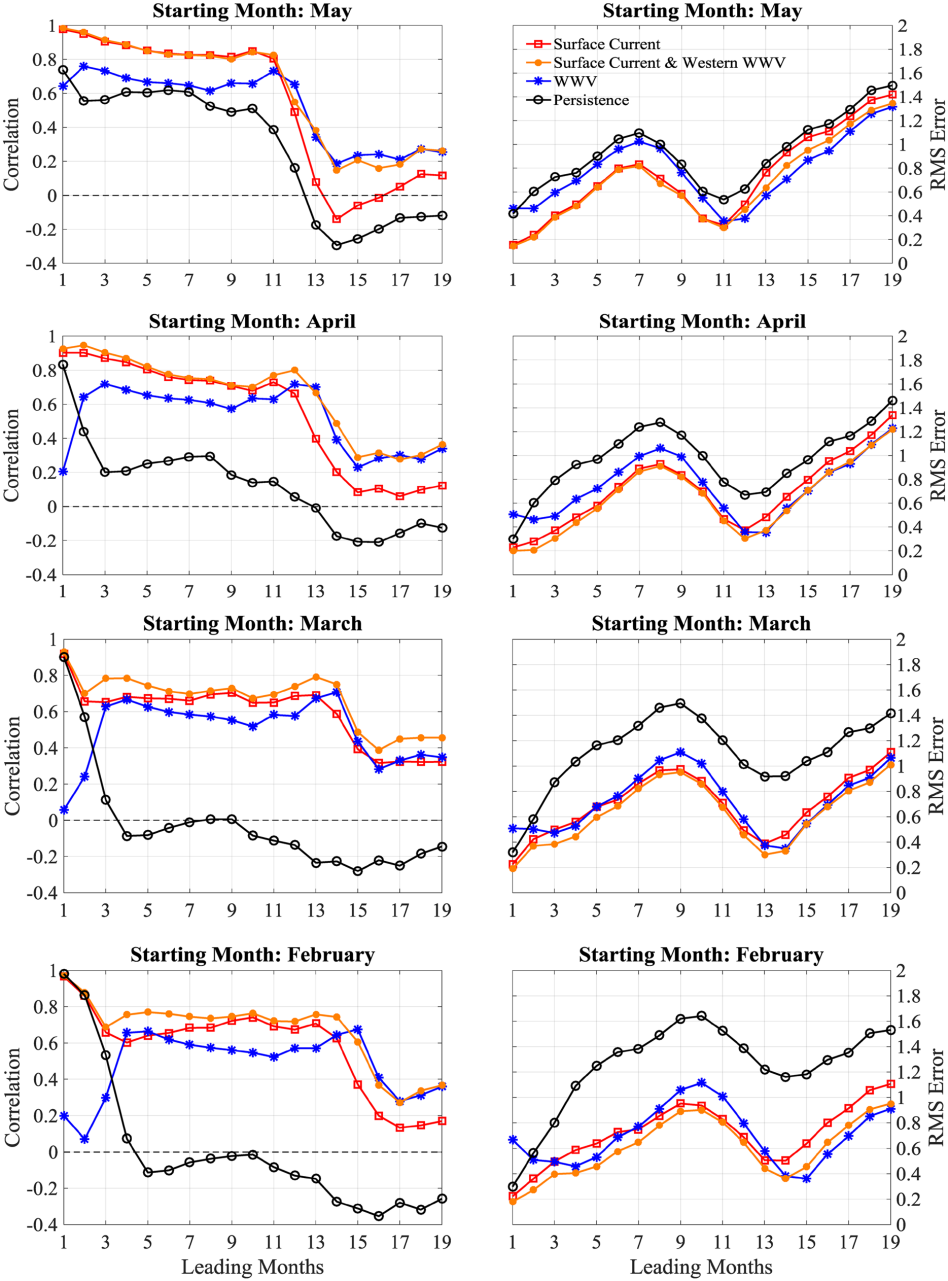
Skills of retrospective predictions of the Niño 3.4 index as a function of lead months. Skills are measured by correlation (left) and rms error (right) between the predicted and observed monthly times series of the Niño 3.4 index. Predictions are initialized in (from top to bottom) May, April, March, and February, respectively, based on surface currents (red), WWV (blue), both surface current and western WWV (orange), and persistence (black). Both the training and prediction periods are from 1993 to 2015. The figure is plotted using MATLAB R2015b (http://www.mathworks.com/).

The surface current in the hotspot identified from the prediction of *N* ^*7*^ also shows high forecasting skill in the subsequent months after July. Figure 6 shows the skills of retrospective predictions during 1993–2005 as a function of lead months, based on surface currents, the WWV and persistence (Methods). The skill is quantified by the correlation coefficient between the predicted Niño 3.4 index against the corresponding observations, and the root mean square of their difference (rms error). A prediction with the correlation being larger than 0.6 is referred to as being skillful^22^. Predictions are initialized in May, April, March and February, respectively. Predictions based on surface current beat the persistence with all lead months. The SPB is evident as indicated by the significant reduction of skills at lead months of 11, 12, 13 and 14, respectively, for predictions started in May, April, March and February. Prior to the approaching to the “barrier” of next year (in April), predictions based on surface currents are all skillful, with correlations being 28%, 21%, 13% and 21% higher, and the rms error being 26%, 16%, 8% and 8% lower, than the predictions based on the WWV on average, for initialization in May, April, March and February, respectively. If the surface current and the western WWV are both used, the prediction skill can be improved, especially for initialization made in March and February. This further indicates that prediction based on surface current has a better ability to overcome the SPB than that based on the WWV alone.

**Figure 7:**
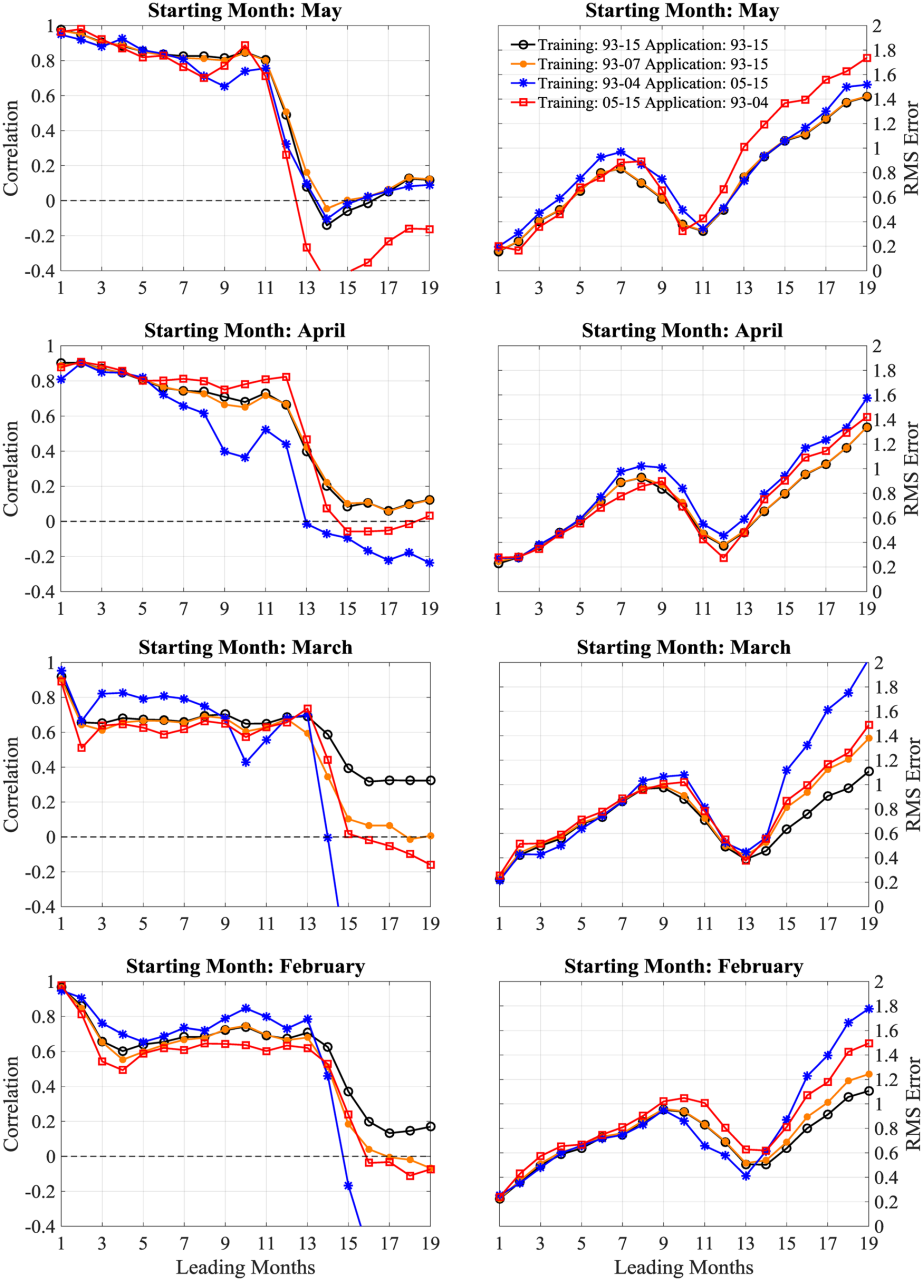
Skills of retrospective predictions of the Niño 3.4 index as a function of lead months, with varying training and application periods. All predictions are made based on surface currents. Skills are measured by correlation (left) and rms error (right) between the predicted and observed monthly times series of the Niño 3.4 index. Predictions are initialized in (from top to bottom) May, April, March, and February, respectively. The training (application) periods are from 1993–2015 (1993–2015) (black), 1993–2007 (1993–2015) (orange), 1993–2004 (2005–2015) (blue), and 2005–2015 (1993–2004) (red), respectively. The figure is plotted using MATLAB R2015b (http://www.mathworks.com/).

Next, we perform cross validations by examining whether the prediction skill based on surface current varies with the training and application periods. Figure 7 compares the skills of four regression trials. The reference trial sets the training and application spanning the same period of 1993–2015. The next trial sets the training period as 1993–2007 and the application period as 1993–2015. Very similar skill is achieved as the reference trial. The other two trials set non-overlapping training and application periods, one period being 1993–2004 and the other being 2005–2015. For these two trials, their differences with the reference trial in terms of correlation and rms error are generally within 0.1 before approaching the SPB of next year. An exception is the trial with training period of 1993–2004 and application period of 2005–2015, when initialized in April the correlation drops below 0.6 at lead times beyond 8 months. Overall, for the latter three trials with training periods different from application periods, their prediction skills are not seriously degraded compared to the reference trial that has an overlapping period for training and application. These cross-validation tests suggest that after trained with existing date, the surface currents-based model can indeed be skillful for prediction.

In summary, the skill of ENSO prediction across the SPB can be improved using surface currents in the southern hotspot. The importance of surface currents in the very localized region, in terms of the overall westward accumulation of warm water, may be related to its significant vertical extension. The satellite ocean current product used in the analysis represents currents averaged from surface to 30 m depth^23^. Furthermore, observations have shown that the SEC here can extend to about 300 m depth^24–26^. Quantification of the anomalous zonal heat transport in this area requires detailed knowledge of the time-space variations in currents and ocean temperature. In this study, the statistical relationship and prediction model are derived from analysis of 23-year observations from 1993 to 2015. It remains to be verified whether this prediction model can be applied over longer durations. Also, it is desirable to apply this model to real-time ENSO predictions^27^. However, it is worth noting that the prediction model based on surface current precursor shows skill both before and after 2002, while predictions based on other precursors, including the WWV and winds^6^, show reduced skill after 2002.

## Methods

### Prediction Based on Regression Relationship

High influence regions of surface current are identified from the prediction of *N* ^*7*^ through a linear multiple regression analysis. The regression model is formulated as *N* ^*7*^-*N* ^*t*^ = *αU* ^*i*−*t*^ + *βV* ^*i*−*t*^ + *ε*, where *N* ^*7*^ and *N* ^*t*^ are the Niño 3.4 index in month numbers 7 (July) and *t*; (*U* ^*i*−*t*^, *V* ^*i*−*t*^) denote surface current anomalies averaged from month *i* to *t*; *α* and *β* are regression coefficients, and *ε* is the residual. Thus, 7-*t* defines the lead time (in months) of the prediction, and *t*-*i* defines the length (in months) to average the surface currents as the prediction precursor. The values of *i* are selected among *t*, *t*-*1*, and *t*-*2*, corresponding to averages lengths of 1, 2 and 3 months. For each choice of *t* and *i*, the location of the “hotspot” where *N* ^*7*^-*N* ^*t*^ and *αU* ^*i*−*t*^ + *βV* ^*i*−*t*^ have the maximum correlation is identified. Table 1 lists the locations of the identified hotspot, and the correlation (*r*) between the observed and predicted *N* ^*7*^ using different precursors with different lead times.

The prediction for *N* ^*t*+*lead*^ is formulated as *N* ^*t*+*lead*^ = *N* ^*t*^ + *αU* ^*i*−*t*^ + *βV* ^*i*−*t*^ + *γ* ${W}_{W}^{t}$ + *δU* ^*i*−*t*^ ${W}_{W}^{t}$ + *ζV* ^*i*−*t*^ ${W}_{W}^{t}$ + *ε*, where (*U* ^*i*−*t*^, *V* ^*i*−*t*^) now denote surface currents in the southern hotspot identified from the prediction of *N* ^*7*^; ${W}_{W}^{t}$ is the western WWV; *lead* denotes the lead time in month. Values of *α*, *β*, *γ*, *δ* and *ζ* are obtained through regression, and some of them can be pre-set to zero to exclude the related precursors. The combination of surface current with the western WWV achieves a higher prediction skill than using the total WWV (*W*) alone. Except for the initial condition (*N* ^*t*^), the other terms may be interpreted as various “precursors”: the displacement of the mean WWV by the anomalous surface currents (*αU* ^*i*−*t*^ + *βV* ^*i*−*t*^); the displacement of the anomalous western WWV by the mean surface currents (*γ* ${W}_{W}^{t}$); and the displacement of the anomalous western WWV by the anomalous surface currents (*δU* ^*i*−*t*^ ${W}_{W}^{t}$ + *ζV* ^*i*−*t*^ ${W}_{W}^{t}$). The prediction skill is measured by the correlation between the observed and predicted *N* ^*t*+*lead*^ and the root-mean-squared (rms) of their difference. The prediction model based on the WWV is formulated as *N* ^*t*+*lead*^ = *γW* ^*t*^ + *ε*. The prediction skill is measured by the correlation and rms error between *N* ^*t*+*lead*^ and *γW* ^*t*^. Without involving the surface current, a higher prediction skill is achieved using the total WWV instead of the western or eastern WWV.

The persistence prediction assumes that *N* ^*t*+*lead*^ = *N* ^*t*^. The prediction skill is measured by the correlation between *N* ^*t*^ and the observed *N* ^*t*+*lead*^ and the rms of their difference.

### Data Sources

Surface currents are obtained from the satellite altimeter Ocean Surface Current Analysis Real-Time (OSCAR) estimate (http://www.oscar.noaa.gov/). The Niño 3.4 index, representing the SST anomaly averaged over 5°N–5°S and 170–120°W, is down loaded from http://www.cpc.ncep.noaa.gov/data/indices/. The WWV is the heat content of the upper ocean within 5°N–5°S and 120°E–80°W with water temperature greater than 20 °C. The WWV is also split into the western (*W*_*W*_) and eastern (*W*_*E*_) parts, defined for regions within 120°E–155°W and 155°W–80°W, respectively. The WWV data are obtained from http://www.pmel.noaa.gov/elnino/. Wind stress data are obtained from the European Centre for Medium-Range Weather Forecasts (ECMWF) ERA-Interim product (http://www.ecmwf.int/en/research/climate-reanalysis/era-interim). SST is obtained from the Hadley Centre Sea Ice and Sea Temperature Dataset (http://www.metoffice.gov.uk/hadobs/hadisst/). Seasonal cycles have been removed from the Niño 3.4 index, and the total, western and eastern WWV. Monthly anomalies of surface current and wind stress are obtained by removing their mean seasonal cycles for the period 1993–2015.
